# Cuticular wax in wheat: biosynthesis, genetics, and the stress response

**DOI:** 10.3389/fpls.2024.1498505

**Published:** 2024-12-03

**Authors:** Ruiyang Tian, Wendi Liu, Yuhai Wang, Wenqiang Wang

**Affiliations:** ^1^ College of Life Sciences, Zaozhuang University, Zaozhuang, China; ^2^ National Key Laboratory of Wheat Improvement, College of Agronomy, Shandong Agricultural University, Tai’an, China; ^3^ Jinan Key Laboratory of Biological Breeding, Spring Valley Agriscience Co., Ltd., Jinan, China

**Keywords:** wheat, cuticular wax, biosynthesis, genetics, stress response

## Abstract

All terrestrial plants possess a hydrophobic cuticle in the outermost layer of their aerial organs that is composed of cutin and wax. The cuticle serves as the first barrier between the plant and the surrounding environment and plays a key role in the resistance of plants to abiotic and biotic stressors. Additionally, they are closely associated with plant growth and development. Cuticular wax has attracted considerable attention as the main mediator of cuticular functions. In this review, we summarize the advances in the research investigating wheat cuticular wax, focusing on three aspects that include biosynthesis, genetics, and stress responses. Additionally, we discuss the applications of cuticular wax in wheat breeding.

## 1 Introduction

Wheat (*Triticum aestivum*), the primary grain crop worldwide, accounts for one-fifth of the total calories consumed by humans (Apples et al., 2018). The United Nations predicts that the global population will reach 9.3 billion by 2050 and surpass 10 billion by 2059. Therefore, food security is a significant global challenge (Lee, 2011; United Nations, 2022). Wheat cultivation is limited by multiple abiotic and biotic stressors that directly affect yield and quality (Song et al., 2024). For example, salt stress affects 20% of the world’s cultivated soils (Arora, 2019) and can lead to wheat yield losses of up to 45% (Ali et al., 2009), reduce the number of tillers (Abbas et al., 2013), and decrease the spikelet and grain weights (Frank et al., 1987). A 40% water reduction may result in a 20.6% loss in wheat yield. The threat of drought stress to wheat has been exacerbated by global warming (Lesk et al., 2016; Hickey et al., 2019). High- and low-temperature environments that occur due to climate change instability also affect wheat yield and quality (Jacott and Boden, 2020). Wheat stripe rust (WS) is a common disease caused by *Puccinia striiformis* f. sp. *Tritici* (*Pst*) that affects up to 4 million hectares of wheat annually in China (Zhang et al., 2019; Chen et al., 2022). Wheat powdery mildew is a widespread disease caused by *Blumeria graminis* f. sp. *Tritici* (*Bgt*) and accounts for approximately 5% of annual wheat yield loss (Savary et al., 2019; Xie et al., 2020). Therefore, considering the influence of abiotic and biotic stress factors, strategies must be developed to cope with high yields, resistance, and quality through genetic improvement.

Approximately 480 to 360 million years ago, ancient algae began to grow on land and became the first land plants (Kenrick and Crane, 1997; Bowman, 2022). These plants faced significant environmental challenges during their growth such as water deficiency, ultraviolet radiation, physical damage, and pathogenic infections (Kong et al., 2020a). In response to abiotic and biotic stresses, plants have evolved hydrophobic cuticles comprising an inner cutin polyester matrix and an outer layer of wax (Samuels et al., 2008; Yeats and Rose, 2013; Renault et al., 2017; Lee et al., 2020). As the supporting structure of the cuticle, cutin is a three-dimensional net polymeric structure composed of ω-hydroxyl groups, an intermediate chain, and C16 and C18 fatty acids and derivatives including hydroxy acids, dicarboxylic acids, and others (Nawrath, 2006; Jetter and Kunst, 2008). Wax is bluish-white (glaucous) in color and primarily composed of very-long-chain fatty acids (VLCFAs), their derivatives, triterpenoids, and certain secondary metabolites (Jetter and Kunst, 2008). The wax is divided into inner and outer epidermal wax in the cuticle. The inner epidermal wax was filled with a net structure formed by a cutin polyester matrix. In contrast, the outer epidermal wax covers the outermost layer of the cuticle and forms a layer of waxy crystals (Jetter and Kunst, 2008). As the dominant contributor to cuticular function, cuticular wax has attracted increasing attention (Kunst et al., 2006). In this review, we focus on the study of cuticular wax in wheat and discuss its future use in genetics and breeding.

## 2 Cuticular wax composition and its biosynthesis pathway in wheat

**Figure 1:**
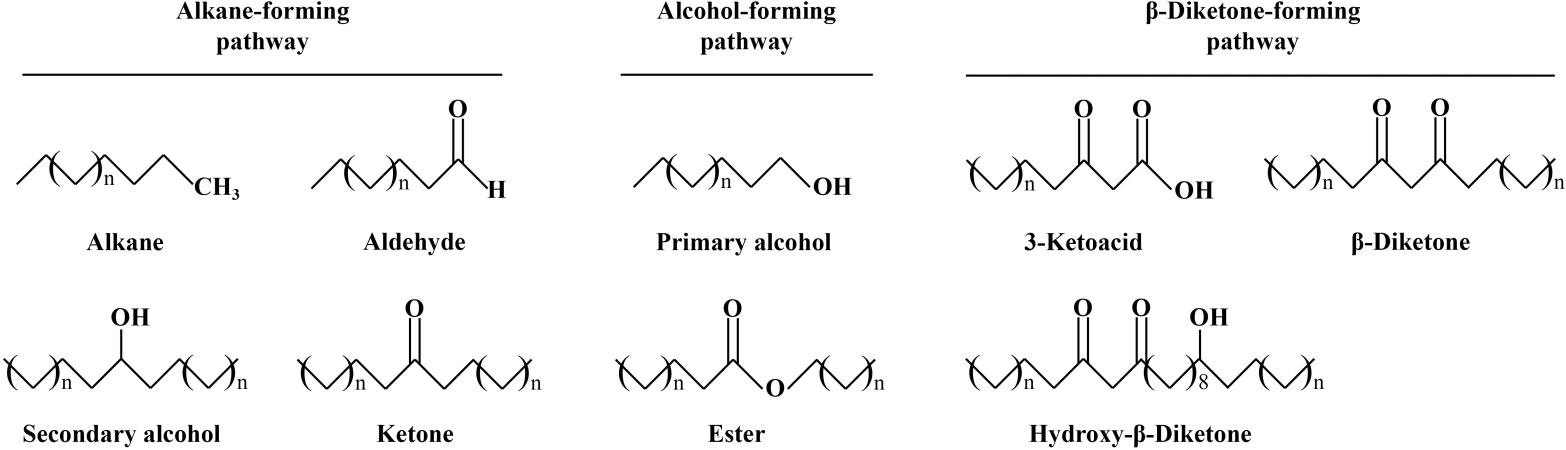
The cuticular wax is composed of many components in wheat. The role of different components depends on their chemical structure and properties.

The cuticular wax of most plants is composed of VLCFAs and their derivatives (**Figure 1**) such as alkanes, alcohols, ketones, and aldehydes (Buschhaus and Jetter, 2011; Xue et al., 2017). However, wax composition varies from plant to plant and even from organ to organ. For example, β-diketone, the main component of wheat wax, is absent in *Arabidopsis* wax, and alkanes are lower in the waxes of corn and barley, while the waxes of soybean and alfalfa are richer in alkanes (Bergman et al., 1991; Post-Beittenmiller, 1996; Hen-Avivi et al., 2016). In addition to interspecies differences, cuticular wax differs across growth and developmental periods and even among different growing environments such as those characterized by distinct temperature and light conditions (Geyer and Schönherr, 1990; Domínguez et al., 2011; Xue et al., 2017). This indirectly indicates that different plants evolved wax biosynthesis genes under specific conditions.

In wheat, cuticular wax content and composition exhibited significant differences in different organs, stages, and environmental conditions. The wax of wheat leaves is predominantly composed of primary alcohol, the wax of wheat flag leaves is primarily composed of alcohol and β-diketone, and the wax of the leaf sheath, stem, and spike is primarily composed of β-diketone (Adamski et al., 2013; Zhang et al., 2013; Wang et al., 2015b). Wax at the wheat seedling stage is composed of fatty alcohols, whereas that at the adult plant stage is composed of alkanes (Yang et al., 2017). The cuticular wax content and composition also exhibit dynamic changes under different environmental conditions. When water is deficient, β-diketone accumulation will increase wax content to prevent water evaporation (Kuruparan et al., 2024). After pest infection, several wax biosynthesis-related genes are induced to enhance single-component accumulation and avoid further damage (Kosma et al., 2010).

Many physiological functions of cuticular wax biosynthesis have been conserved throughout the evolution of different plants (Kramer and Havens, 2009; Kong et al., 2020a; McWhite et al., 2020; Wang and Chang, 2022). Most conceptions of the wax biosynthesis pathway are based on research investigating model plants such as *Arabidopsis*. In recent years, the cuticular wax biosynthesis pathway in wheat has been gradually clarified based on studies involving *Arabidopsis*. For example, *AtSHN1* was the first identified transcription factor involved in cuticular wax biosynthesis in *Arabidopsis* (Aharoni et al., 2004). As the homolog of *AtSHN1*, *TaSHN1* has been identified in wheat (Bi et al., 2018). Similarly, the overexpression of *TaSHN1* also altered wax accumulation in the cuticle, and the alkane content was higher in bread wheat (Bi et al., 2018).

**Figure 2:**
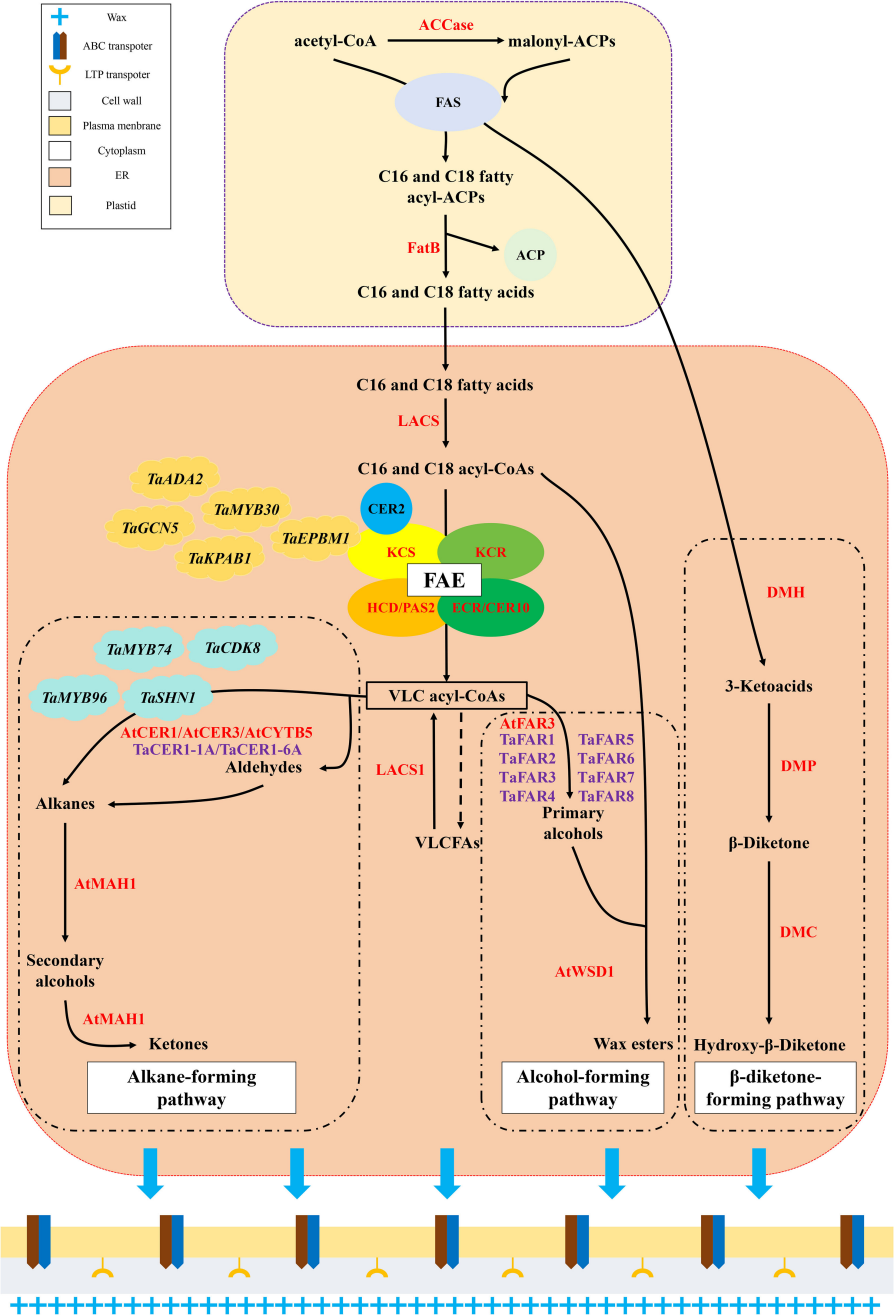
The pathway of cuticular wax biosynthesis in plants. The alcohol-forming pathway and alkane-forming pathway were ubiquitous in C3 and C4 plants. The β-diketone-forming pathway is only confirmed in C3 plants, and it was unique in wheat and other *Triticeae* Dumort plants.

Wax biosynthesis involves several metabolic pathways and protein complexes. It can be roughly divided into the following three steps that include synthesis of wax precursors (C16 and C18 fatty acids), synthesis of VLCFA acyl-CoAs, and synthesis, processing, and transport of wax derivatives (**Figure 2**; **Table 1**).

**Table 1:** The related wax biosynthesis gene loci and function in wheat.

Gene	Chromosome	Function	Reference
*W1*	2BS	*W1* affects β-diketone synthesis in wheat, and it is homologous to *Cer-cqu* in barley	(Tsunewaki and Ebana, 1999; Wu et al., 2013; Allen and Vogel, 1960)
*W2*	2DS	Unknown	
*W3*	2BS	Unknown	(Zhang et al., 2015)
*W4*	3DL	Unknown	(Bi et al., 2016)
*W5*	7DL	Unknown	(Li et al., 2020)
*GLOSSY1*	2DS	Unknown	(Li et al., 2021)
*IW1*	2BS	*IW1* produces a microRNA miRW1, which targets *W1-COE* (Carboxylesterase) to suppress the glaucousness phenotype	(Bi et al., 2018; Kong and Chang, 2018)
*IW2*	2DS	Unknown	(Discoll and Jensen, 1964; Tsunewaki, 1962)
*IW3*	1BS	Unknown	
*TaSHN1/WIN1*	Unknown	Overexpression of *TaSHN1* increased alkanes in leaves, knockdown of it reduced aldehydes and alkanes on blades.	(Bi et al., 2018; Kong and Chang, 2018)
*TaMYB74*	Unknown	*TaMYB74* binds to the MYBR1 and MYBR2 *cis*-elements of *TaSHN1*.	(Bi et al., 2016)
*TaEPBM1*	Unknown	Binding protein of the *TaECR* and enhanced *TaECR* expression levels.	(Kong et al., 2020b)
*TaMYB96*	Unknown	Targeting the motif “CAACCA” of three key wax biosynthesis genes, *TaCER1-6A*, *TaCER1-1A*, and *TaFAR4*	(He et al., 2022)
*TaMYB30*	Unknown	*TaMYB30* can directly bind to *TaKCS1* and *TaECR* and positively accelerate wax biosynthesis.	(Liu et al., 2023)
*TaKPAB1*	Unknown	*TaKPAB1* directly binds to the *TaKCS6*, knockdown of *TaKPAB1* reduces cuticular wax deposition.	(Wang et al., 2019)
*TaCDK8*	Unknown	*TaCDK8* can facilitate *TaSHN1* transcription and regulate cuticular wax biosynthesis in wheat.	(Kong and Chang, 2018)
*TaADA2*	Unknown	The complex stimulates *TaECR* expression through histone modification.	(Kong et al., 2020b)
*TaGCN5*	Unknown		
*TaCER1-1A*	1A	Overexpression of *TaCER1-1A* changes the cuticular wax composition	(Li et al., 2019)
*TaCER1-6A*	6A	Overexpression of *TaCER1-6A* induced cuticular wax component accumulation	(He et al., 2022)
*TaFAR1*	Unknown	In wheat, several FAR-like proteins have been shown to participate in the synthesis of primary alcohols, which are specifically involved in the production of C22 to C30 very long chain primary alcohols.	(Wang et al., 2015a)
*TaFAR2*	Unknown		(Wang et al., 2016)
*TaFAR3*	Unknown		
*TaFAR4*	Unknown		
*TaFAR5*	Unknown		(Wang et al., 2015b)
*TaFAR6*	Unknown		(Chai et al., 2018)
*TaFAR7*	Unknown		
*TaFAR8*	Unknown		

Acetyl-CoA produces acyl–acyl carrier proteins (malonyl-ACPs) in the plastids of epidermal cells via the carboxylation of acetyl-CoA carboxylase (ACCase) and transacylation of acyl carrier protein (ACP) (Byers and Gong, 2007; Li-Beisson et al., 2013). Malonyl-ACP is a two-carbon donor involved in the synthesis of C16 and C18 fatty acyl ACPs. Subsequently, acetyl-CoA generates C16 and C18 fatty acyl-ACPs under the catalytic action of fatty acid synthetase (FAS) multi-enzyme complexes (Ohlrogge and Browse, 1995; Günenc et al., 2022). C16 and C18 fatty acyl-ACPs are hydrolyzed by fatty acyl–acyl ACP thioesterase B (FatB) to release ACPs and free C16 and C18 fatty acids (Li-Beisson et al., 2013). Finally, free C16 and C18 fatty acids are exported to the endoplasmic reticulum (ER) for the second stage of wax biosynthesis (Samuels et al., 2008).

The fatty acid elongase (FAE) complex plays a decisive role in the second stage of wax biosynthesis. It is composed of four different enzymes that include β-ketoacyl-CoA synthase (KCS), β-ketoacyl-CoA reductase (KCR), β-hydroxyacyl-CoA dehydratase (HCD/PAS2), and β-enoyl-CoA reductase (ECR/CER10) (Kim et al., 2022). After entering the ER, free C16 and C18 fatty acids are esterified to C16 and C18 acyl-CoA by long-chain acyl-CoA synthetase (LACS) proteins (Li-Beisson et al., 2013). Subsequently, C16 and C18 fatty acyl-CoAs undergo condensation, reduction, dehydration, and re-reduction cycles catalyzed by multimeric FAE complexes (Lee et al., 2009; Kim et al., 2022). In this cycle, an acyl-CoA precursor (C16 and C18 fatty acyl-CoA) and malonyl-CoA undergo a condensation reaction catalyzed by KCS to form β-ketoacyl-CoA. β-ketoacyl-CoA is then reduced to generate β-hydroxy acyl-CoA, and this is catalyzed by KCR. Subsequently, β-hydroxy acyl-CoA loses an H_2_O molecule under the catalysis of HCD/PAS2 to produce enoyl-CoA. ECR/CER10 catalyzes the reduction of enoyl-CoA to acyl-CoA. In each cycle, the final acyl-CoA is two carbons longer than the primary acyl-CoA precursor until it extends to very-long-chain acyl-CoAs (VLC acyl-CoAs) of > 20 carbons (Lee et al., 2009; Kunst and Samuels, 2009; Wang et al., 2017; Bai et al., 2022; Kim et al., 2022). Once the carbon chain length exceeds 28, the involvement of CER2-LIKE proteins in the BAHD superfamily of acyltransferases is indispensable (Haslam et al., 2015; Haslam and Kunst, 2021). To modify the chain length, CER2-LIKE proteins interact with KCS to adjust the chain length specificity of the elongase complex (Haslam et al., 2015; Haslam and Kunst, 2021). When VLC acyl-CoAs are produced, they are hydrolyzed by a hypothetical VLC acyl-CoA thioesterase to release VLCFAs (Lewandowska et al., 2020). A small proportion of VLCFAs may be released directly into the cuticular wax or reactivated back to VLC acyl-CoAs by LACS1. However, most VLC acyl-CoAs are further modified in the ER to synthesize wax derivatives (Kunst and Samuels, 2003; Samuels et al., 2008).

Wax derivatives can be produced via either alcohol-forming or alkane-forming pathways (**Figure 2**; **Table 1**). In the alcohol-forming pathway, most VLC acyl-CoAs are catalyzed by fatty acyl-CoA reductase 3 (FAR3) to produce primary alcohols (Wen and Jetter, 2009). In wheat, several FAR-like proteins have been demonstrated to participate in the synthesis of primary alcohols that are specifically involved in the production of C22–C30 very-long-chain primary alcohols (Wang et al., 2015a, 2015b, 2016; Chai et al., 2018). Wax synthetase/diacylglycerol acyltransferase 1 (WSD1) catalyzes the binding of primary alcohols to acyl-CoAs to form wax esters (Tomiyama et al., 2017; Li et al., 2008). Other derivatives, including aldehydes, alkanes, secondary alcohols, and ketones, were synthesized via the alkane-forming pathway. As vital products of this pathway, alkanes can be synthesized via direct generation by VLC acyl-CoAs (Jenks et al., 1995) or indirect generation, whereby VLC acyl-CoAs are first oxidized to aldehydes and then reduced to alkanes (Jenks et al., 1995). Both pathways are affected by an alkane synthesis protein complex comprising ECERIFERUM1 (CER1), CER1-LIKE1, CER3, and cytochrome B5 (Pascal et al., 2019). Two CER1 proteins have been identified in wheat. Both proteins are closely involved in the alkane-forming pathway and significantly affect alkane accumulation in wheat cuticular wax (Li et al., 2019; He et al., 2022). Moreover, alkanes can be hydroxylated to secondary alcohols by the CYP96A family cytochrome P450 enzyme that acts as a mid-chain alkane hydroxylase (MAH1), producing ketones via the same reaction (Greer et al., 2007). These wax constituents are transported from the ER to the plasma membrane through the combined action of ATP-binding cassette transporters and lipid-transfer proteins through the cell wall to the cell cuticle, where they undergo self-assembly to form wax crystals (Pighin et al., 2004; Rees et al., 2009; Wong et al., 2019).

Wheat, as a member of the Triticeae Dumort family, exhibits another parallel crucial wax biosynthesis pathway responsible for β-diketone biosynthesis in addition to the two main wax biosynthesis pathways discussed above (**Figure 2**; **Table 1**). This pathway was first proposed in genetic studies of barley and later confirmed in wheat (Hen-Avivi et al., 2016; Wettstein-Knowles, 1995; Schneider et al., 2016). In this pathway, the β-diketone biosynthesis gene cluster that comprises three genes, diketone metabolism polyketide synthase (*DMP*), diketone hydrolase/carboxylesterase (*DMH*), and diketone cytochrome P450 (*DMC*), plays a predominant role (Hen-Avivi et al., 2016). The β-diketone biosynthesis pathway synthesizes β-diketone and related derivatives (Post-Beittenmiller, 1996). Based on previous studies of the β-diketone biosynthesis pathway, 3-ketoacyl-ACP produced in the FAS multienzyme complexes are first captured by DMH (Wettstein-Knowles, 1995; Zhang et al., 2013; Hen-Avivi et al., 2016). In the ER, 3-ketoacyl-ACP hydrolysis catalyzed by DMH results in the release of 3-ketoacid that is then converted to β-diketone and a hydroxylated derivative by the successive actions of DMP and DMC (Wettstein-Knowles, 1995; Zhang et al., 2013; Hen-Avivi et al., 2016). Finally, β-diketone and hydroxy-β-diketone are transported to the cuticle to assemble cuticular wax (Wettstein-Knowles, 1995; Zhang et al., 2013; Hen-Avivi et al., 2016).

A recent study has revealed the β-diketone-forming pathway in barley (Sun et al., 2023). As a core intermediate, 3-ketoacid was initially formed in the β-diketone-forming pathway through DMH. Sun et al. (2023) analyzed the origin and formation of the functional group of β-diketone and finally presented the head-to-head condensation hypothesis. An interesting catalytic reaction occurs during the DMP reaction. There was recarboxylative reaction to catalyze 3-ketoacids and fatty acyl-CoAs for head to head condensation into β-diketones (Sun et al., 2023). When β-diketones were formed, most of them would be transported to cuticular layer, while some would participate in the next DMC catalyzed reaction to produce hydroxy-β-diketones (Sun et al., 2023).

The β-diketone-forming pathway is different from the other two ubiquitous biosynthesis pathway. There are multiple enzymatic reactions in the other two pathways, while the β-diketone-forming pathway with only three enzymes can synthesize multiple wax components. Although a recent study was performed in barley (Sun et al., 2023), the details of β-diketone-forming pathway remain obscure in wheat. When the 3-ketoacids biosynthesis process begins, DMH must capture the intermediate 3-ketoacid-ACP. This action appears to compete with the production of C16 and C18 fatty acyl-ACPs and indirectly affects the other two ubiquitous biosynthesis pathways. Therefore, we speculate that β-diketone-forming pathway needs to be closely regulated. Moreover, as mentioned above, β-diketone is a major component of cuticular wax in wheat, but it is absent from the cuticular wax of the model plant *Arabidopsis* (Hen-Avivi et al., 2016). This may indicate that the wax biosynthesis pathway in *Arabidopsis* lacks genes encoding enzymes that catalyze the reaction to synthesize β-diketone or influence by other factors.

## 3 Genetic and regulatory mechanism of cuticular wax biosynthesis in wheat

**Figure 3:**
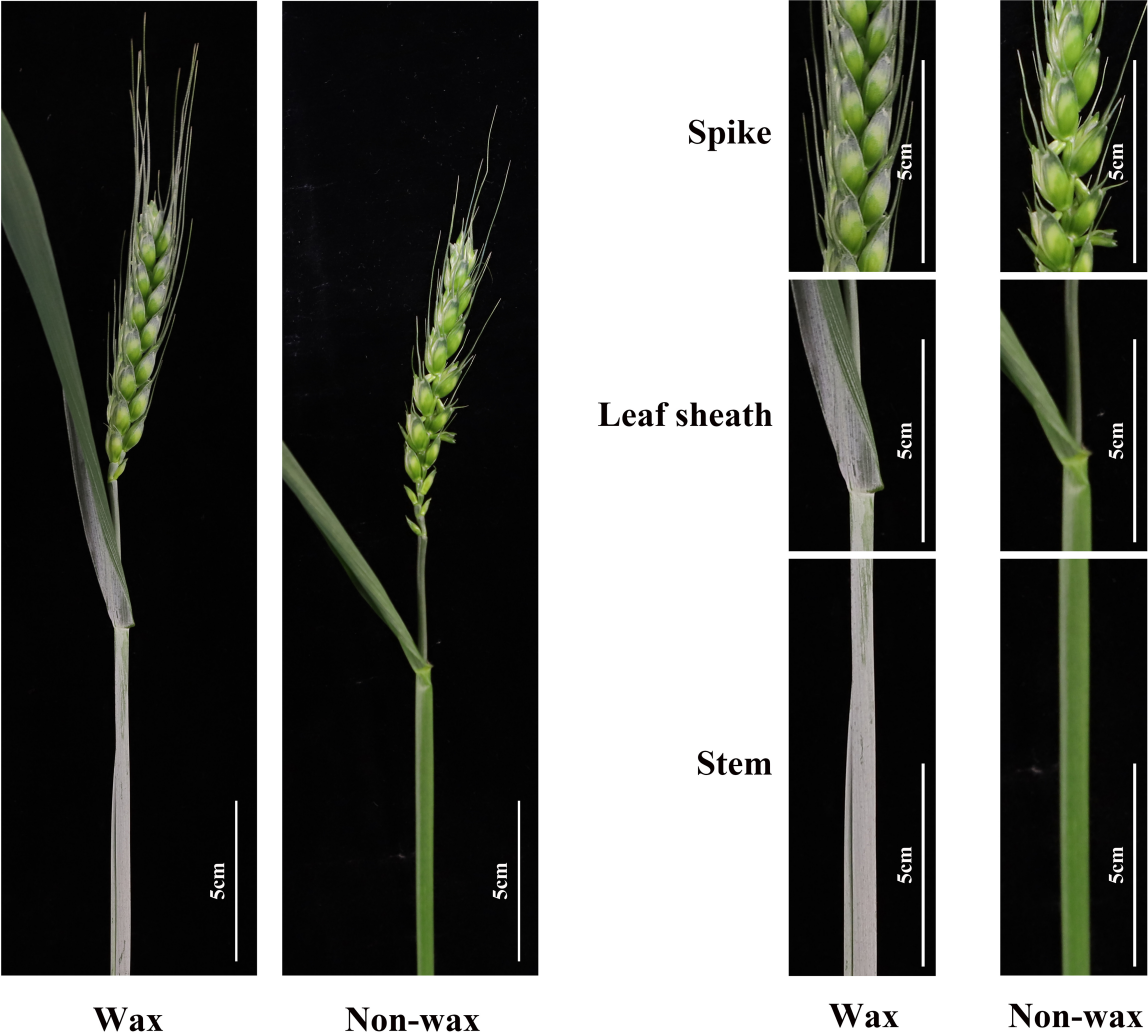
We utilized the wheat cultivar ‘Jimai38’ to create a mutant population in the previous study and investigated the wax-deficient phenotype of wheat at heading stages. The representative phenotypes of wax and wax-deficient plants were presented at single tiller, spike, leaf sheath, and stem.

Most commercial bread wheat contains wax on the surfaces of organs such as leaves, stems, and spikes (**Figure 3**), and glaucousness was observed after flowering. With the continuous in-depth study of cuticular wax over the last few decades, its genetic basis and regulatory mechanisms have gradually been revealed.

### 3.1 Genetic basis of cuticular wax biosynthesis in wheat

The glaucousness trait of cuticular wax in wheat is controlled by two sets of dominant genes that include wax production loci (*W1* and *W2*) and wax inhibition loci (*Iw1* and *Iw2*) (Tsunewaki and Ebana, 1999). Genetic linkage analysis of *W1* and *Iw1* demonstrated that they are located on the short arm of chromosome 2BS and are closely linked at a genetic distance of 2 cM (Wu et al., 2013). *W2* and *Iw2* reside on the short arm of chromosome 2DS at a genetic distance of at least 130 cM (Tsunewaki and Ebana, 1999; Wu et al., 2013). *W1*, *W2*, *Iw1*, and *Iw2* are the key genes that control cuticular wax biosynthesis (Allen and Vogel, 1960; Tsunewaki, 1962; Discoll and Jensen, 1964; Tsunewaki, 1964; Tsunewaki and Ebana, 1999; Wu et al., 2013). The dominant glaucousness production genes, *W1* and *W2*, appear simultaneously or separately to produce a glaucousness phenotype. In contrast, the inhibition loci for glaucousness (*Iw1* and *Iw2*) exhibit a dominant effect (Tsunewaki, 1962; Wu et al., 2013), as the presence of *Iw1*, *Iw2* (or both) can inhibit the glaucousness phenotype (Tsunewaki, 1962; Wu et al., 2013). Schneider et al. identified three diverse *cer* genes on the short arm of barley chromosome 2H. *Cer-c*, *-q* and *-u* are tightly linked, forming a gene cluster known as *Cer-cqu* (Schneider et al., 2016). In wheat, the *W1* locus is a gene cluster that affects β-diketone synthesis and is homologous to *Cer-cqu* in barley (Hen-Avivi et al., 2016; Schneider et al., 2016). The orthologs of *Cer-cqu* are *W1-COE* (DMH), *W1-PKS* (DMP), and *W1-CYP* (DMC) (Huang et al., 2017). As a miRNA precursor gene, *Iw1* produces the miRNA *miRW1* that targets the cleavage sites of *W1-COE* (DMH) to suppress the glaucousness phenotype (Huang et al., 2017).

In addition to these key wax-related genes, numerous other genes affecting wax traits have been identified. Zhang et al. (2015) discovered a wax-deficient mutant in the wheat cultivar ‘Bobwhite’ and identified a new gene involved in β-diketone synthesis on chromosome 2BS, designated *W3*. Nishijima et al. cloned *W4* from *Aegilops tauschii Coss* (D genome progenitor) located on chromosome 3DL (Nishijima et al., 2018). Li et al. disclosed a wax-deficient mutant *W5* from the wheat cultivar ‘Jimai22’ and finely mapped it to a 194-kbp region on chromosome 7DL (Li et al., 2020). Similarly, another wax-affected gene, *GLOSSY1*, was identified and finely mapped to a 308.1-kbp region on chromosome 2DS (Li et al., 2021). *Iw3*, a new tissue-specific wax-inhibition locus, has been mapped to chromosome 1BS in Emmer wheat (Wang et al., 2014).

### 3.2 Regulatory mechanism of cuticular wax biosynthesis in wheat

Transcription factors (TFs) play important roles in the regulation of cuticular wax biosynthesis. SHINE1/WAX INDUCER1 (*SHN1*/*WIN1*) was the first TFs associated with wax regulation in *Arabidopsis*. *AtSHN1* contains a highly conserved APETALA2 (AP2) domain (Aharoni et al., 2004). *AtSHN1* overexpression drives the expression of a series of genes in *Arabidopsis*, such as *CER1*, *KCS1*, and *CER2*, that produce more wax (Broun et al., 2004). Moreover, *SHN1* directly targets the promoter of *LACS2* and modifies cuticle permeability (Kannangara et al., 2007). *TaSHN1*/*WIN1* is a typical TF of the SHN1 family and is a homolog of *AtSHN1* (Bi et al., 2018). The overexpression of *TaSHN1* influences the components of cuticular wax and significantly increases alkane levels in leaves (Bi et al., 2018). Additionally, the knockdown of *TaWIN1* expression weakened the accumulation of very-long-chain aldehydes and alkanes on wheat blade surfaces (Kong and Chang, 2018).

Previous studies have reported that Myeloblastosis (MYB) TFs are precisely regulated during wax biosynthesis in many plant species (Lee and Suh, 2015, 2022). Several MYB TFs such as TaMYB74, TaEPBM1, TaMYB96, and TaMYB30 have been demonstrated to regulate wax biosynthesis in wheat (Bi et al., 2016; Kong et al., 2020b; He et al., 2022; Liu et al., 2023). *TaMYB74* specifically binds to the MYBR1 and MYBR2 *cis*-elements of the wax biosynthesis-related gene *TaSHN1* under drought stress (Bi et al., 2016). TaEPBM1, an R2R3-type MYB TF, was isolated as a binding protein for *TaECR* that enhances *TaECR* expression levels (Kong et al., 2020b). *TaMYB96*, was observed to target the conserved motif “CAACCA” of three key wax biosynthesis genes, *TaCER1-6A*, *TaCER1-1A*, and *TaFAR4* (He et al., 2022). Recently, *TaMYB30*, a novel MYB TF, was isolated from wheat (Liu et al., 2023). Similar to its homologous gene, *AtMYB30* modulates VLCFA synthesis (Raffaele et al., 2008). *TaMYB30* can directly bind to *TaKCS1* and *TaECR* and accelerate wax biosynthesis (Liu et al., 2023). The basic helix-loop-helix (bHLH) transcription factor family regulates plant cuticle development (Wu et al., 2011; Li et al., 2016). *TaKPAB1* binds directly to *TaKCS6* and recruits *TaCHR729* (Wang et al., 2019). Moreover, knockdown of *TaKPAB1* reduces cuticular wax deposition in wheat (Wang et al., 2019).

Cuticular wax biosynthesis is also influenced by other pathways. Cyclin-dependent kinase 8 (CDK8) is a critical component of the eukaryotic mediator complex. In *Arabidopsis*, CDK8 interacts with *AtSHN1* to regulate cuticle development, and a *cdk8* mutant exhibits a notably different cuticle structure (Zhu et al., 2014). As a homolog of *AtCDK8*, *TaCDK8* interacts with *TaSHN1* to facilitate *TaSHN1* transcription and regulate cuticular wax biosynthesis in wheat (Kong and Chang, 2018). Interestingly, a strong phosphorylation signal was detected in the immunocomplex kinase assay, demonstrating that TaCDK8-mediated phosphorylation increased the transcription-activating role of TaSHN1 (Kong and Chang, 2018). TaADA2-TaGCN5 histone acetyltransferase (HAT) complex regulates cuticular wax biosynthesis in wheat. The wheat TF TaEPBM1 can directly interact with the TaADA2-TaGCN5 HAT complex. This complex stimulates *TaECR* expression through histone modification, thus promoting the biosynthesis of cuticular wax components (Kong et al., 2020b).

## 4 Cuticular wax responses to abiotic and biotic stresses in wheat

As the first primary physical barrier, the cuticle plays an indispensable role in plant responses to abiotic and biotic stressors. Specifically, cuticular wax can limit non-stomatal water loss and protect plants from other stresses such as UV radiation, high temperatures, pathogens, and pests (Li et al., 2019; Long et al., 2003; Djemal and Khoudi, 2021; Skamnioti and Gurr, 2007; Zhou and Zhang, 2020). Previous studies have demonstrated that cuticular wax plays an important role in abiotic and biotic stresses tolerance in wheat (**Table 2**).

**Table 2:** The cuticular wax biosynthesis genes involved in abiotic and biotic strress in wheat.

	Stress Type	Gene	Genotypes	Function	Reference
Abiotic Stress	Drought	*TaSHN1/WIN1*	AP2/ERF family TF	Overexpression of *TaSHN1* increased alkanes in leaves and reduced the stomatal density to enhance drought tolerance.	(Bi et al., 2016)
		*TaCER1-1A*	Aldehyde decarbonylase in decarbonylation pathway	Overexpression of *TaCER1-1A* changes the cuticular wax composition and conferred drought resistance.	(Li et al., 2019)
		*TaCER1-6A*	Aldehyde decarbonylase in decarbonylation pathway	Overexpression of *TaCER1-6A* induced cuticular wax component accumulation and reduced cuticle permeability and reinforced drought tolerance.	(He et al., 2022)
	Salinity	Unknown			
	Hot	Unknown			
	Cold	Unknown			
Biotic Stress	Pathogen	*TaSHN1/WIN1*	AP2/ERF family TF	BSMV-VIGS induced the silencing of *TaCDK8* and *TaWIN1* resulted in reduced cuticular wax accumulation and repressed *Bgt* germination.	(Kong and Chang, 2018)
		*TaCDK8*	A component of eukaryotic mediator complex		
		*TaECR*	Enoyl-CoA reductase	The TaEPBM1-TaADA2-TaGCN5 protein complex stimulates *TaECR* expression, caused the reduction of cuticular wax and the germination of *Bgt*.	(Kong et al., 2020b)
		*TaEPBM1*	R2R3-type MYB TF		
		*TaADA2*	Alteration/deficiency in activation-2		
		*TaGCN5*	General control nonderepressible 5		
		*TaKCS6*	3-Ketoacyl CoA synthase	*TaKPAB1* binds to the E-box cis-element of TaKCS6 and recuit *TaCHR729*, induced the reduction of cuticular wax deposition and *Bgt* conidia germination.	(Wang et al., 2019)
		*TaCHR729*	CHD3 type chromatin remodeling factor		
		*TaKPAB1*	bHLH type TF		
	Pest	Unknown			

### 4.1 Cuticular wax responses to abiotic stresses in wheat

Drought, cold, heat, and salinity stresses are major environmental factors that affect the growth and development of wheat and threaten food security (**Table 2**). These environmental factors have become increasingly frequent due to climate change (Fedoroff et al., 2010; Zhu, 2016).

Heterologous overexpression of *TaCER1-1A* causes changes in the cuticular wax composition and confers significant drought resistance in *Arabidopsis* and rice (Li et al., 2019). Overexpression of *TaSHN1* and *TaCER1-6A* induces cuticular wax accumulation, reduces cuticle permeability, and reinforces drought tolerance (Bi et al., 2018; He et al., 2022). Recently, eight *FAR*-like genes (*TaFAR1*, *TaFAR2*, *TaFAR3*, *TaFAR4*, *TaFAR5*, *TaFAR6*, *TaFAR7*, and *TaFAR8*) were demonstrated to be involved in cuticular wax biosynthesis (Wang et al., 2015a, 2015b, 2016; Chai et al., 2018). Transcriptional expression analysis revealed that *TaFAR1*, *TaFAR2*, *TaFAR3*, *TaFAR4*, *TaFAR5*, *TaFAR6*, *TaFAR7*, and *TaFAR8* were induced under drought and cold stress. *TaFAR2*, *TaFAR3*, and *TaFAR4* were positively regulated by salinity stress, and the expression levels of *TaFAR6*, *TaFAR7*, and *TaFAR8* were significantly increased by heat stress (Wang et al., 2015a, 2015b, 2016; Chai et al., 2018). These results suggest that *FAR*-like genes are associated with cuticular wax biosynthesis and actively respond to multiple abiotic stressors.

### 4.2 Cuticular wax responses to biotic stresses in wheat

The cuticle is the first interface between the plant and the external environment and protects plants from pathogens and pests (Wang et al., 2020; Chen, 2021). Many studies have confirmed that pathogen or pest invasion can affect the expression of cuticular wax biosynthesis genes that regulate immune responses in wheat (**Table 2**).

Wheat powdery mildew caused by *Bgt* is a devastating disease that reduces global wheat yield (Sánchez-Martín et al., 2021; Zhu et al., 2022). Several studies have demonstrated that cuticular wax is closely associated with *Bgt* infections (Kong and Chang, 2018; Wang et al., 2019; Kong et al., 2020b). BSMV-VIGS induces *TaCDK8* and *TaWIN1* expression, resulting in reduced cuticular wax accumulation and the repression of *Bgt* germination (Kong and Chang, 2018). Similarly, the knockdown of *TaKCS6*, *TaCHR729*, *TaKPAB1*, *TaECR*, *TaEPBM1*, *TaADA2*, and *TaGCN5* leads to a reduction in cuticular wax and germination of *Bgt* (Wang et al., 2019; Kong et al., 2020b). The hessian fly is a wheat pest that causes annual global crop losses (Smiley et al., 2004). After infestation of wheat with Hessian flies, the total amount of cuticular wax did not change significantly, more individual wax components were detected, and the transcript levels of *CRE3*, *CER4*, and *KCS6* genes involved in wax synthesis were significantly upregulated in resistant plants (Kosma et al., 2010). These results suggest that cuticular wax plays an important role in the compatible and incompatible interactions between plants and pests, particularly in the permeability of the cuticle.

## 5 Perspectives on the role of cuticular wax in breeding applications

Germplasm resources are critical for breeding and genetic improvements (Liu et al., 2012). Creating mutant materials using chemical, physical, and biological methods is a reliable strategy for generating germplasm resources (Wang et al., 2024). Over the years, wax-deficient mutants have provided valuable genetic resources for mapping wax biosynthesis loci such as *W3*, *W5*, and *GLOOSY1* in wheat (Zhang et al., 2015; Li et al., 2020, 2021). This remains the mainstream technique for exploring new wax sources using mutants. The application strategies include identification of wax mutant phenotypes, sequencing of trait-associated mutations (STAM) to acquire candidate genes (Ni et al., 2023), and molecular marker-assisted breeding. Thus, the “phenotype- STAM -molecular marker” model is considered as an effective engine for genetic improvement of wax traits.

We propose a rapid technological system for wheat breeding to improve the application efficiency of wax traits. This system was developed based on molecular marker selection, genome-wide liquid SNP chip development, and haploinduction technology. Taking the wax-dominant gene *W1* as an example, the first step was to select a material containing the target gene *W1* and an excellent wheat variety without wax traits. Second, *W1*-specific Kompetitive Allele-Specific PCR (KASP) markers were designed, and a genome-wide liquid SNP chip was developed for excellent wheat varieties. The two materials were then grown in a greenhouse. Considering the effect of breeding applications, the hybrid generation was backcrossed with the excellent wheat variety for to 3-4 generations. During backcrossing, the wax trait was investigated to identify the *W1* gene of each hybrid generation. When the background of the hybrid generation recovered to approximately 95% of that of the excellent wheat variety, haploid induction was performed on the backcrossed generation to obtain homozygous lines. Finally, the wax phenotypes and agronomic traits were verified in the field. This system combines traditional breeding and genomic technologies.

**Figure 4:**
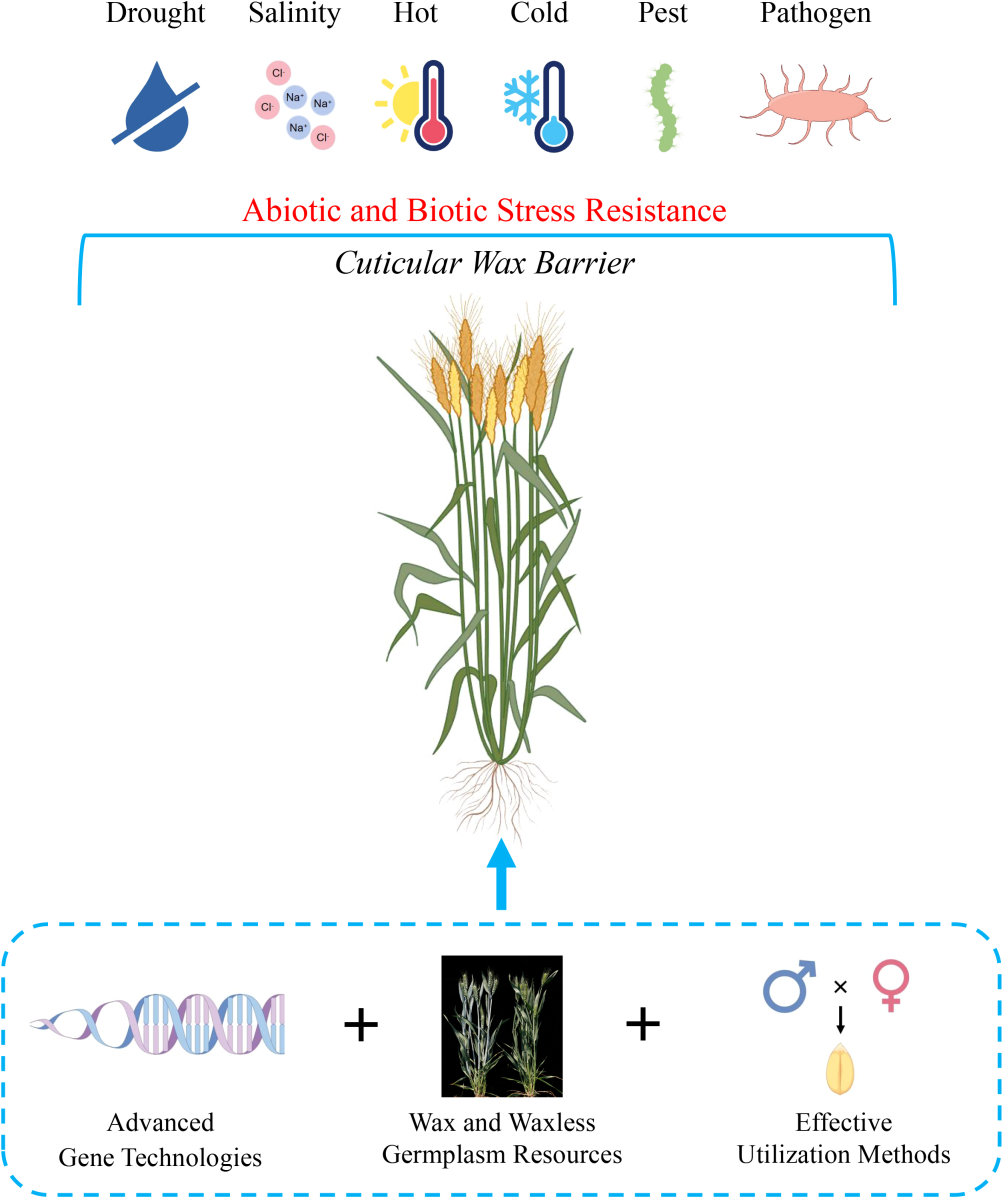
We purposed a model for future cuticular wax genetic improvement in wheat. As a protective barrier, the cuticular wax will confer a positive effect for wheat to withstand abiotic and biotic stress. Three key points were discussed in this review, including advanced gene technologies, wax and waxless germplasm resources, and effective utilization methods. The combination of three key points will accelerate application and development of wax traits in the context of genetic improvement. In addition to wax traits, the model can also be applied on other useful traits. The figure was created by figdraw.com.

From the perspective of natural evolution, cuticular wax plays an important role in combating biotic and abiotic stress. Therefore, it is necessary to cultivate the protective traits of waxes in crops. Future research should focus on three primary directions to support the breeding of effective cuticular waxes in wheat, including advanced genetic technologies, various wax germplasm resources, and effective utilization methods (**Figure 4**).

## 6 Concluding remarks

In this review, we summarize recent research advances in wheat cuticular wax. Cuticular wax is a natural protective film that functions as a critical barrier in various stressful environments. Over the past few decades, remarkable progress has been made in the study of cuticular waxes. However, due to limited knowledge, the specific roles of cuticular wax components remain unclear, and the differences in wax composition between different species or organs require further investigation. Moreover, the regulatory mechanisms of cuticular wax biosynthesis and stress responses warrant further studies to deepen our understanding and improve the utilization efficiency of cuticular wax to enhance stress resistance in wheat.
